# V144D Mutation of* SPTLC1* Can Present with Both Painful and Painless Phenotypes in Hereditary Sensory and Autonomic Neuropathies Type I

**DOI:** 10.1155/2018/1898151

**Published:** 2018-10-18

**Authors:** Kwo Wei David Ho, Nivedita U. Jerath

**Affiliations:** Department of Neurology, University of Florida, USA

## Abstract

Hereditary sensory and autonomic neuropathy type I (HSAN I) is an autosomal dominant disease characterized by distal sensory loss, pain insensitivity, and autonomic disturbances. The major underlying causes of HSAN I are point mutations in the SPTLC1 gene. Patients with mutations in the SPTLC1 genes typically exhibit dense sensory loss and incidence of lancinating pain. Although most of these mutations produce sensory loss, it is unclear which mutations would lead to the painful phenotype. In this case series, we report that the V144D mutation in SPTLC1 gene may relate to both painful and painless peripheral neuropathies. The unique clinical phenotype of this mutation may guide clinical workup and treatment for patients with painful and painless neuropathies.

## 1. Introduction

HSAN I is an autosomal dominant disease characterized by distal sensory loss, pain insensitivity and autonomic disturbances. The major underlying causes of HSAN I are mutations in the SPTLC1 gene, which encodes the first subunit of serine palmitoyltransferase (SPT). These mutations result in the formation of atypical neurotoxic sphingolipids and subsequent dysfunction in the peripheral nerves [1]. Multiple mutations in SPTLC1, including p.C133W, p.C133Y, p.C133R, p.V144D, p.S331F, pA310G, p.S331Y, and p.A352V have been found to cause HSAN I [2]. Although painless neuropathy and lancinating pain have both been described in cases of HSAN I [3, 4], it is unclear how each mutation corresponds to painful or painless phenotypes. In this case series, we report that the V144D mutation in SPTLC1 gene can produce both painful and painless neuropathies.

## 2. Case Presentation

### 2.1. Case 1

A 37-year-old woman presented with intense pain in her feet while walking. She began to feel water droplets burning through her feet at age 29. Her symptoms continued to progress to an intense burning and lightening-like pain while walking, as if her feet were scraped by sandpaper and then dipped in rubbing alcohol. The pain was so severe that she thought about cutting her feet off.

**Figure 1:**
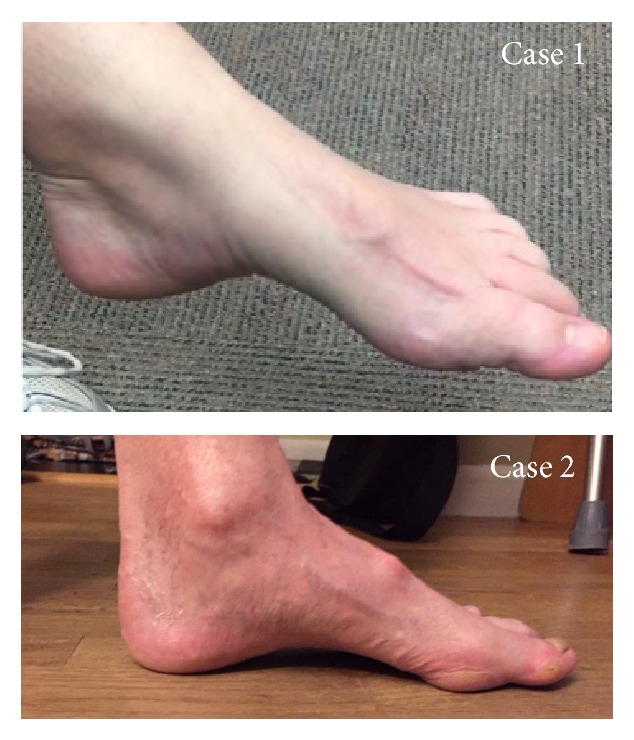
Feet of the presented cases: both presented with high arches.

Examination was significant for severe pain in her feet; a simple touch was equivalent to a “bowling ball dropped on her skin”. She had high arched feet on exam (Figure 1, Case 1). There was decreased sensation to pinprick and light touch up to her ankle. Vibratory sense was decreased up to her knees. She was unable to walk on her heels and reflexes were absent. She had full strength throughout. Her Charcot-Marie-Tooth examination score was a 10 out of 28 [5]. Electromyography and nerve conduction studies showed evidence of chronic axonal neuropathy with normal nerve conduction velocities and absent sural and peroneal responses. Sequencing of 72 neuropathy genes [6] showed a pathogenic variant, c.431T>A (p.Val144Asp) of the SPTLC1 gene.

### 2.2. Case 2

A 74-year-old male presented with generalized numbness. He started to have significant numbness in his legs in his forties, which progressed to above his knees. At the time of visit, he had no sensation in his hands, fingers, and toes. His legs would jump when he lied down at night and he had ankle pain when he walked. He had cramps in his thighs, calves, and left arm. He had some hearing difficulties and tremors. Family history was significant for the daughter of the subject carrying the same mutation; she also had similar symptoms of generalized numbness. The subject also has two unaffected daughters and a sister who were offered genetic testing but they declined.

On exam, he had absent light touch and pinprick sensation below his knees and elbows. He had reduced vibratory sense below his knees. He was areflexic throughout. Strengths were 4+/5 in his hands and full strength in his legs. There was atrophy of the bilateral feet and hands, and he could not walk tandem, on toes or on heels. He had high arches bilaterally and his feet could not be easily brought into a neutral position (Figure 1, Case 2). His Charcot-Marie-Tooth examination score was 13/28. Electromyography and nerve conduction studies showed evidence of chronic axonal neuropathy with normal nerve conduction velocities and absent sural, peroneal, and tibial responses. Sequencing of 72 neuropathy genes [6] showed a pathogenic variant, c.431T>A (p.Val144Asp) of the SPTLC1 gene.

## 3. Discussion

Common genetic polymorphisms have been shown to affect the development and perception of pain [7, 8]. Rare, single-gene mutations causing painful phenotype are less common [9, 10]. HSAN I is a single-gene disorder classically characterized by painless sensory loss. Painful phenotype in this disorder has not been genetically well-described. This is the first report to show that the V144D mutation of SPTLC1 can cause both painless and painful neuropathies. The V144D variant in the SPTLC1 gene has been previously reported in association with HSAN I [4]. Patients with this mutation usually have sensory loss, but the painful phenotype has never been reported with this particular mutation. In the original papers where SPTLC1 mutations were found to be a cause of HSAN1, dense sensory loss and incidence of lancinating pain were described as typical symptoms [3, 4]. However, it was unclear which mutations would lead to lancinating pain and which would lead to sensory loss. The only mutation that was clearly documented to be associated with painful neuropathy was the C133W mutation of SPTLC1 [11]. This present report is important in that it adds an additional mutation in SPTLC1 which may cause painful polyneuropathy. It is possible that all mutations in SPTLC1 can cause both painful and painless polyneuropathies, but more data is needed to support such an assumption. This case series underscores the importance of genetic testing to diagnose HSAN in patients with both painful and painless neuropathies.
